# Supplementary material for: An open-source framework for large-scale, flexible evaluation of biomedical text mining systems
Source: J Biomed Discov Collab. 2008 Jan 29;3:1. doi: 10.1186/1747-5333-3-1 (PMC2276192; doi:10.1186/1747-5333-3-1)
Supplement: Additional file 1 — Evaluation platform source code. The additional file contains the software evaluation framework discussed in this paper. The most current release can be downloaded from SourceForge.net [1]. See the accompanying README file for instructions on installation and use. Also included in the distribution is a collection of UIMA wrappers for some commonly used BioNLP tools and annotated corpora. [file 1747-5333-3-1-S1.zip › bionlp_uima_repository-1.0.1/docs/api/src-html/edu/uchsc/ccp/uima/ae/nlp/tokenizer/GeniaTaggerOutputFileLoader_AE.html]

```
001    /*
002     *
003     * The Original Code is UCHSC UIMA.
004     *
005     * The Initial Developer of the Original Code is University of Colorado.  
006     * Copyright (C) 2005.  All Rights Reserved.
007     *
008     * UCHSC Utilities was developed by the Center for Computational Pharmacology
009     * (http://compbio.uchsc.edu) at the University of Colorado Health 
010     *  Sciences Center School of Medicine with support from the National 
011     *  Library of Medicine.  
012     *
013     *
014     * Contributor(s):
015     *   William A. Baumgartner <william.baumgartner@uchsc.edu> (Original Author)
016     *   
017     * Created: November 2005
018     * Dependencies:
019     * TODO:
020     * Changes:
021     */
022    package edu.uchsc.ccp.uima.ae.nlp.tokenizer;
023    
024    import java.io.IOException;
025    import java.util.Iterator;
026    import java.util.List;
027    
028    import org.apache.uima.analysis_engine.ResultSpecification;
029    import org.apache.uima.analysis_engine.annotator.AnnotatorConfigurationException;
030    import org.apache.uima.analysis_engine.annotator.AnnotatorContext;
031    import org.apache.uima.analysis_engine.annotator.AnnotatorContextException;
032    import org.apache.uima.analysis_engine.annotator.AnnotatorInitializationException;
033    import org.apache.uima.analysis_engine.annotator.AnnotatorProcessException;
034    import org.apache.uima.analysis_engine.annotator.JTextAnnotator_ImplBase;
035    import org.apache.uima.jcas.JCas;
036    
037    import edu.uchsc.ccp.uima.annotation.CCPDocumentInformation;
038    import edu.uchsc.ccp.uima.util.UIMA_Util;
039    import edu.uchsc.ccp.util.external.geniatagger.GeniaTaggerOutputFileParser;
040    import edu.uchsc.ccp.util.nlp.annotation.TextAnnotation;
041    
042    /**
043     * This class loads in a file which contains GENIA tagger output.
044     * 
045     * @author William A. Baumgartner, Jr.
046     * 
047     */
048    public class GeniaTaggerOutputFileLoader_AE extends JTextAnnotator_ImplBase {
049    
050        private final boolean DEBUG = false;
051    
052        public final String PARAM_GENIA_TAGGER_OUTPUT_FILE = "GeniaTaggerOutputFile";
053    
054        private GeniaTaggerOutputFileParser gtofp;
055    
056        @Override
057        public void initialize(AnnotatorContext ac) throws AnnotatorInitializationException, AnnotatorConfigurationException {
058            /* get the filename for the pretagged mutations file (.pprj) from the descriptor file */
059            String geniaTaggerOutputFile = null;
060            try {
061                geniaTaggerOutputFile = (String) ac.getConfigParameterValue(PARAM_GENIA_TAGGER_OUTPUT_FILE);
062            } catch (AnnotatorContextException e1) {
063                e1.printStackTrace();
064            }
065    
066            /* parse the pretagged mutations file and load the HashMap */
067            try {
068                gtofp = new GeniaTaggerOutputFileParser(geniaTaggerOutputFile);
069            } catch (IOException e) {
070                e.printStackTrace();
071            }
072            super.initialize(ac);
073        }
074    
075        public void process(JCas jcas, ResultSpecification arg1) throws AnnotatorProcessException {
076            /* Object object should be a CASInitializerArguments object */
077    
078            /* get the documentID */
079    
080            String documentID = "-1";
081            Iterator docInfoIter = jcas.getJFSIndexRepository().getAnnotationIndex(CCPDocumentInformation.type).iterator();
082            if (docInfoIter.hasNext()) {
083                Object possibleDocInfo = docInfoIter.next();
084                if (possibleDocInfo instanceof CCPDocumentInformation) {
085                    CCPDocumentInformation ccpDocInfo = (CCPDocumentInformation) possibleDocInfo;
086                    documentID = ccpDocInfo.getDocumentID();
087                } else {
088                    error("Expecting CCPDocumentInformation but got: " + possibleDocInfo.getClass().getName());
089                }
090            }
091    
092            String documentText = jcas.getDocumentText();
093    
094            if ((documentText != null) && (gtofp.hasNext(documentText))) {
095                List<TextAnnotation> textAnnotations = gtofp.next();
096    
097                if (textAnnotations != null) {
098                    /* check to make sure documentIDs correspond */
099                    if (textAnnotations.size() > 0) {
100                        if (!documentID.equals(textAnnotations.get(0).getDocumentID())) {
101                            error("Document ID mismatch!!!  expecting: " + documentID + " but retrieved: "
102                                    + textAnnotations.get(0).getDocumentID());
103                        }
104                    }
105                    UIMA_Util uimaUtil = new UIMA_Util();
106                    uimaUtil.putTextAnnotationsIntoJCas(jcas, textAnnotations);
107                }
108            } else {
109                error("Expecting parser output in file, however none was found for document: " + documentID);
110            }
111    
112        }
113    
114        private void error(String message) {
115            System.err.println("ERROR -- GeniaTaggerOutputFile_AEr: " + message);
116        }
117    
118        private void warn(String message) {
119            System.err.println("WARNING -- GeniaTaggerOutputFile_AEr: " + message);
120        }
121    
122        private void debug(String message) {
123            if (DEBUG) {
124                System.err.println("DEBUG -- GeniaTaggerOutputFile_AEr: " + message);
125            }
126        }
127    
128    }
```
